# Comparative Physiology and Psychology

**Published:** 1885-03

**Authors:** 


**Comparative Physiology and Psychology.** A Discussion of the Evolution and Selections of the Mind and Body of Man and Animals. By L. V. Clevenger, M. D., Special Pathologist County Insane Asylum, Chicago. Chicago: Jansen McClurg & Co. 1885.

The author states that some of the original ideas contained in this book have appeared in scientific and medical publications during the past five years as the papers were presented to scientific societies. He has condensed these papers and thoroughly revised them, and presents them to the scientists. The work thus offered bears evidences of the closest study and work upon the part of its author, and he has succeeded in presenting a most valuable contribution in comparative physiology and anatomy. We commend the work to physiologists.

